# pSILAC mass spectrometry reveals ZFP91 as IMiD-dependent substrate of the CRL4^CRBN^ ubiquitin ligase

**DOI:** 10.1038/ncomms15398

**Published:** 2017-05-22

**Authors:** Jian An, Charles M. Ponthier, Ragna Sack, Jan Seebacher, Michael B. Stadler, Katherine A. Donovan, Eric S. Fischer

**Affiliations:** 1 Department of Cancer Biology, Dana-Farber Cancer Institute, Boston, Massachusetts 02215, USA; 2 Department of Biological Chemistry and Molecular Pharmacology, Harvard Medical School, Boston, Massachusetts 02215, USA; 3 Friedrich Miescher Institute for Biomedical Research, Maulbeerstrasse 66, CH-4058 Basel, Switzerland; 4 Swiss Institute of Bioinformatics, CH-4058 Basel, Switzerland

## Abstract

Thalidomide and its derivatives lenalidomide and pomalidomide (IMiDs) are effective treatments of haematologic malignancies. It was shown that IMiDs impart gain-of-function properties to the CUL4-RBX1-DDB1-CRBN (CRL4^CRBN^) ubiquitin ligase that enable binding, ubiquitination and degradation of key therapeutic targets such as IKZF1, IKZF3 and CSNK1A1. While these substrates have been implicated as efficacy targets in multiple myeloma (MM) and 5q deletion associated myelodysplastic syndrome (del(5q)-MDS), other targets likely exist. Using a pulse-chase SILAC mass spectrometry-based proteomics approach, we demonstrate that lenalidomide induces the ubiquitination and degradation of ZFP91. We establish ZFP91 as a *bona fide* IMiD-dependent CRL4^CRBN^ substrate and further show that ZFP91 harbours a zinc finger (ZnF) motif, related to the IKZF1/3 ZnF, critical for IMiD-dependent CRBN binding. These findings demonstrate that single time point pulse-chase SILAC mass spectrometry-based proteomics (pSILAC MS) is a sensitive approach for target identification of small molecules inducing selective protein degradation.

## Abstract (web summary)

Targeting therapeutically-relevant proteins for degradation is an emerging paradigm in drug discovery. Here the authors describe a sensitive pulse SILAC mass spectrometry-based proteomics approach that reports global changes in protein stability following drug treatment in a single time point experiment.

The ubiquitin proteasome system (UPS) constantly remodels the proteome to regulate a plethora of cellular processes^1^,^2^,^3^. In the UPS, ubiquitin is covalently coupled to a substrate lysine by activity of an E1 (ubiquitin activating enzyme), E2 (ubiquitin conjugating enzyme) and E3 (ubiquitin ligase) enzyme cascade. Specificity in the UPS is largely conferred by E3 ligases, of which there are more than 600 in the human genome^4^.

Thalidomide and its derivatives lenalidomide and pomalidomide (collectively known as IMiDs for immune-modulatory drugs), while initially marketed as sedatives found to cause severe teratogenic side effects, are widely used to treat haematologic malignancies, including multiple myeloma (MM) and 5q deletion associated myelodysplastic syndrome (del(5q) MDS)^5^,^6^,^7^,^8^. Lenalidomide binds to the ubiquitin ligase CRL4^CRBN^ and exhibits dual activity by inhibiting CRL4^CRBN^ from ubiquitinating endogenous substrates such as MEIS2 (refs 9, 10), while simultaneously promoting the CRL4^CRBN^-dependent ubiquitination and degradation of IKZF1 (Ikaros), IKZF3 (Aiolos) and Casein kinase 1 alpha (CSNK1A1)^10^,^11^,^12^,^13^,^14^. IKZF1 and IKZF3 have been implicated in MM anti-proliferative effects of IMiDs, and CSNK1A1 has been shown to be the efficacy target of lenalidomide in del(5q)-MDS^8^. Recent reports have further demonstrated that lenalidomide disrupts the ubiquitin-independent regulation of CD147 and MCT1 by CRBN^15^ and that glutamine synthetase (GS) is ubiquitinated by CRL4^CRBN^ in an IMiD-independent fashion^16^. However, the pleiotropic effects of IMiDs and the largely unexplained adverse effects such as the profound teratogenicity and neurotoxicity suggest that other substrates likely exist. To identify novel CRL4^CRBN^ substrates regulated by lenalidomide, we performed proteomics studies in non-hematopoietic cell lines to avoid masking of results with the dominant presence of IKZF1/3 in hematopoietic tissues.

Mass spectrometry-based methods have become standard for the identification of E3 ubiquitin ligase substrates and were successfully applied to various classes of ligases, such as the Cullin RING ligase family SCF/CRL1 (refs 17, 18, 19). However, other ligase families have proven recalcitrant to commonly used methods such as affinity-purification mass spectrometry, di-Gly proteomics or whole proteome relative quantification. Common complications for the identification of ubiquitin ligase substrates are secondary effects, such as transcriptional response or changes to translation. For profiling of protein stability-directed drugs, such as lenalidomide, most secondary effects can be reduced through relatively short drug treatments (<24 h) compared to the typical treatment times for RNAi or genetic inactivation (typically >48 h). However, faced with the challenge of relatively small changes to total protein levels, we reasoned that an approach directed at measuring changes to protein turnover in response to drug treatment would provide increased sensitivity compared to relative quantification of total protein abundance. Such a sensitive method would also be valuable to a growing field of drug development efforts directed at protein degradation^20^,^21^,^22^,^23^.

Pulse-chase experiments, performed with ^35^S isotope labelling or through blockade of translation by treatment with cycloheximide (CHX), are considered the gold standard method to investigate protein stability. Modern mass spectrometry combined with stable isotope labelling (SILAC) can principally be used to perform pulse-chase experiments on a proteome-wide scale, and this has been applied to interrogate global protein synthesis and decay rates^24^,^25^,^26^,^27^,^28^. However, such experiments typically involve multiple time points.

Here we show that direct measurement of changes in protein stability in a single time point mass spectrometry experiment is a sensitive and robust alternative to identify targets of lenalidomide. The method is based on temporally controlled incorporation of heavy amino acids by changing the growth medium (pSILAC) simultaneously with applying the desired perturbation, such as treatment with a drug. Samples are subjected to shotgun proteomics utilizing liquid chromatography coupled tandem mass spectrometry (LC-MS/MS)^29^. The pre-existing (light amino acid containing) and newly synthesized (heavy amino acid containing) protein species can be differentially quantified based on their characteristic mass difference to derive heavy to light (H/L) protein ratios^30^. In such an experimental design, and assuming constant incorporation rate, logarithmic H/L ratios should increase linearly over time. H/L protein ratios represent a quantitative measure of protein stability and differences between treated and control cells can be used to identify drug-induced changes to protein turnover, such as induced degradation of target proteins. We demonstrate that a single time point pSILAC experiment is sufficient to identify CSNK1A1 along with new substrate candidates. We further show that the zinc finger (ZnF) protein, ZFP91, a putative ubiquitin ligase^31^,^32^, is a *bona fide* lenalidomide-dependent CRL4^CRBN^ substrate.

## Results

### Identification of novel CRL4^CRBN^ targets through pSILAC MS

We sought to explore a single time point pSILAC approach, which would increase the depth of proteome profiling and significantly reduce the required machine time compared to multi time point experiments. To understand the scopes and limitations of this approach, we performed a direct comparison with multi time point pSILAC and quantified the changes to total protein abundance using tandem mass tags (TMT)^33^.

**Figure 1:**
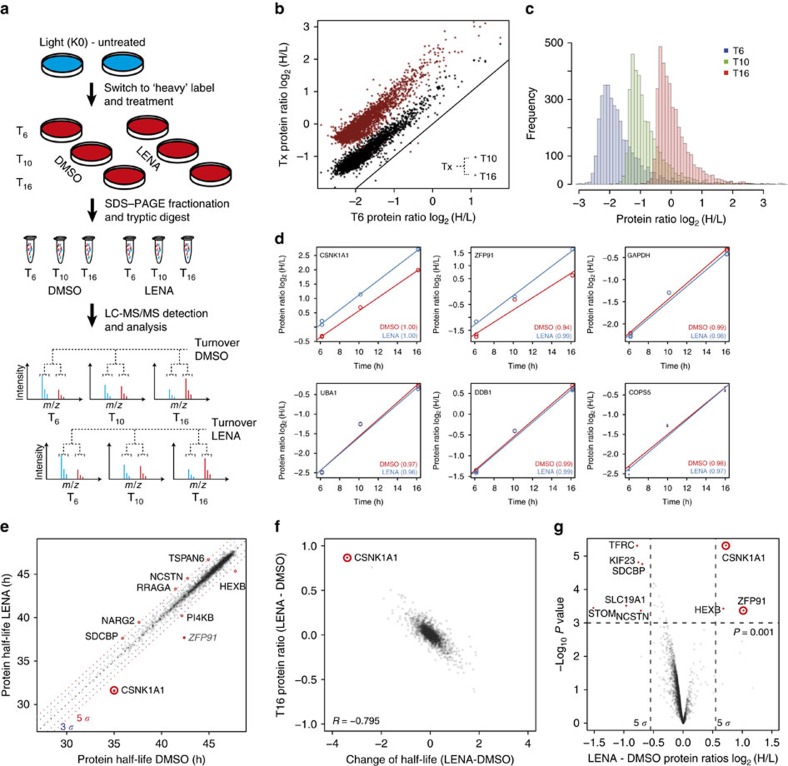
Multi time point pSILAC mass spectrometry. (**a**) Outline of the multi time point pulse-SILAC mass spectrometry experimental design. For simplification, replicate and reverse experiments are not depicted in this figure. (**b**) Scatter plot depicting the change of H/L protein ratios over time. Protein ratios of the 10 and 16 h time points are compared to the 6 h time point. Data in this figure are presented as means of biological replicates for T6 and T16 (*n*=2) or individual data points for T10 (*n*=1). (**c**) Frequencies of H/L protein ratios at different time points (data as in **b**). (**d**) Plots depicting the logarithmic H/L protein ratios over time are shown for the two validated targets CSNK1A1 and ZFP91 as well as for control proteins GAPDH, UBA1, DDB1 and CopS5. Differential turnover for CSNK1A1 and ZFP91 is observed, while stable conditions are found for controls. Data in this figure are presented as individual data points and separate *r*^2^ values for the linear regression are provided for lenalidomide and DMSO samples. (**e**) Scatter plot comparing protein half-lives of lenalidomide treated and control samples. Protein half-lives were obtained by fitting the H/L protein ratios of T6 (*n*=2), T10 reverse (*n*=1) and T16 (*n*=2) time points to a decay function^24^. For further analysis, we retained only proteins quantified in all samples and with *r*^2^>0.9 for both linear regression fits (DMSO and lenalidomide treated), resulting in a total of 2,759 proteins. CSNK1A1 was found to exhibit a reduced half-life in presence of lenalidomide. Blue and red dotted lines indicate ±3 and ±5 s.d., respectively. ZFP91 was dropped from the data analysis for missing values in two replicate measurements. The depicted delta-half-life for ZFP91 is calculated using imputed values from replicates to replace the missing values. (**f**) Scatter plot depicting the negative correlation between differences in protein half-life (data as in **e**), and differences in log_2_ H/L protein ratios at the T16 time point (LENA-DMSO, mean of two biological replicates). *R* corresponds to the Pearson's correlation coefficient. (**g**) Fold change in H/L ratios comparing lenalidomide to DMSO control treatment on the *x* axis (HEK293 T16 SILAC samples). Moderated *t*-test *P* values were calculated using the limma package and shown as −log_10_ values on the *y* axis. The vertical dashed lines indicate ±5 s.d. log_2_ fold change in H/L ratio and the horizontal dashed line indicates *P* value <0.001. Data shown represents two biological replicates.

To generate a reference data set for direct comparison, we first designed a multi time point pSILAC experiment. We used metabolic pulse labelling with heavy amino acids to measure protein turnover in growing HEK293T cells treated with 30 μM lenalidomide or dimethyl sulfoxide (DMSO) control (Fig. 1a). The high concentration of lenalidomide was chosen to achieve maximum response in a short time window and thereby aid substrate identification. HEK293T cells were chosen to bias the experiment towards targets beyond the well-studied transcription factors IKZF1 and IKZF3 that are found explicitly in hematopoietic lineages^34^. Cells were collected in duplicates at three time points, 6 h (T6), 10 h (T10) and 16 h (T16) post treatment with lenalidomide or DMSO and parallel metabolic labelling. To prevent arginine to proline conversion, a pervasive problem in SILAC, the cell culture medium was supplemented with excess unlabelled proline. Label swap experiments at the T6 and T10 time points were performed and analysed to exclude the possibility of SILAC label artefacts (growth medium changing from H/L isotope label on treatment, see also Supplementary Figs 1a–c and 2a–c). While we observe a small systematic offset in H/L protein ratios comparing forward to reverse experiments (most pronounced at early time points), the overall correlation between forward and reverse experiments and random spot checks suggest that label artefacts can be neglected for further analysis. Whole-cell lysates were pre-fractionated into 15 fractions by SDS–PAGE and tryptic peptides subjected to LC-MS/MS analysis on an Orbitrap VELOS platform (see Methods section). Mass spectrometry data were analysed with the MaxQuant software^35^. We identified 100,763 peptide sequences, which were assigned to 6,328 unique protein groups (false discovery rate <1% on peptide and protein level; requiring min one unique peptide quantified for a protein to be included).

Polypeptides synthesized after drug treatment and metabolic labelling will predominantly incorporate heavy amino acids, thus the H/L protein ratios can serve as a direct readout of protein turnover (Fig. 1b–d)^24^. H/L protein ratios were quantile normalized (Supplementary Fig. 2a,b) and showed high correlation coefficients across replicates and time points (around 0.9, Supplementary Fig. 2c), which reflects the expected small overall perturbation induced by the drug, and the consistent rank order of H/L protein ratios over time. Of the 6,328 unique protein groups identified, the 2,837 quantified across all time points and replicates were selected to subsequently calculate protein half-lives as previously described^24^. Additional quality filtering was applied to reject proteins for which the linear regression resulted in *r*^2^<0.9 for a total of 2,759 remaining protein groups (Fig. 1e). Due to the experimental design geared towards drug effects, the shortest time point was set at 6 h post treatment, compromising the ability to quantify very short-lived proteins. We hypothesized that proteins differentially degraded in a lenalidomide-dependent manner should exhibit differences in protein half-lives and would thus be candidate substrates. Comparing global protein half-lives of lenalidomide treated and control cells (Fig. 1e), we identified casein kinase 1 alpha (CSNK1A1) as the most affected protein, which we validated elsewhere^11^ in parallel to a study published by Krönke *et al*.^12^, who linked CSNK1A1 degradation to 5q-MDS efficacy of lenalidomide. Analysis of the full data set confirmed that the effect on CSNK1A1 is more pronounced and clearly identified at the late time point (Fig. 1f), supporting the concept of single time point measurements for target identification. To test the proposed approach of a single time point pSILAC experiment, we analysed the T16 time point (two replicates) alone by calculating moderated *t*-test *P* values using the limma package^36^. We included all protein groups that were identified with at least three unique peptides and quantified with more than two peptides (*n*=2,654). Comparison of the T16 H/L protein ratios of lenalidomide or DMSO-treated samples identifies CSNK1A1, ZFP91 and HEXB as significantly downregulated targets along with a number of proteins whose turnover is reduced on drug treatment (Fig. 1g and Supplementary Table 2).

### Single time point pSILAC mass spectrometry

**Figure 2:**
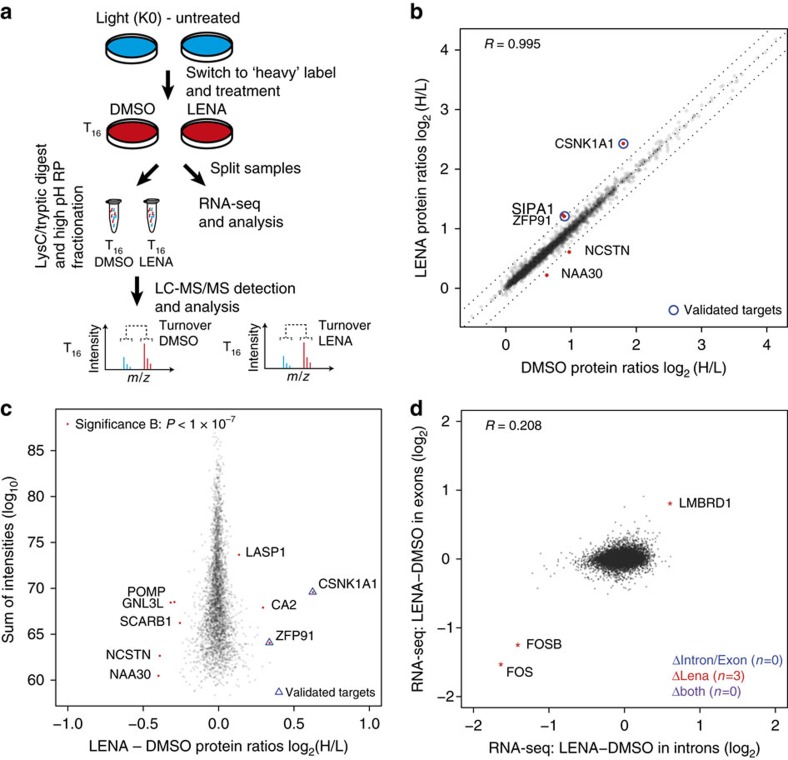
Single time point pulse-SILAC is sufficient to identify drug targets. (**a**) Outline of the single time point pulse-SILAC mass spectrometry and RNA-seq experimental design. (**b**) Scatter plot depicting substrate candidates identified by comparing the H/L protein ratios of lenalidomide treated to DMSO control cells. Data presented are the means of protein ratios (*n*=4 biological replicates and *R* represents the Pearson's correlation coefficient). Only protein groups are shown that were quantified with minimum of three unique peptides in each experiment (3,352). (**c**) Scatter plot depicting the identification of substrate candidates. Log_2_ changes in LENA to DMSO H/L protein ratio are shown on the *x* axis, and log_10_ sum of MS1 intensities (combined for heavy and light peptides) on the *y* axis. Significance B was calculated for 10 intensity bins and proteins with Significance B *P* values <1 × 10^−7^ are shown in red^35^. Only protein groups are shown that were quantified with minimum of three unique peptides in each experiment (3,352). (**d**) Comparison of intronic (*x* axis) and exonic (*y* axis) expression changes in lenalidomide treated to DMSO control cells after 16 h (T16, Hct116), identifies three genes with altered transcription, and none with altered post-transcriptional regulation. *R* indicates the Pearson's correlation coefficient. Data presented are the means of biological replicates (*n*=4).

Next, we sought to assess the robustness of our approach by conducting a single time point experiment in a different cell line (Hct116), with four replicate measurements at 16 h post treatment, of which, two were designed as label swap experiments (see Supplementary Fig. 1a–c). Hct116 cells were treated with either 30 μM lenalidomide or DMSO control concomitant with transfer into medium containing amino acids labelled with heavy isotopes (similar to the first experiment). Cells were collected 16 h post treatment and split for pSILAC MS and RNA-seq experiments (Fig. 2a). Samples for MS analysis were pre-fractionated and subsequent analyses were performed on an Orbitrap Fusion mass spectrometer. LC-MS/MS data were processed with MaxQuant^35^, identifying 108,546 peptide sequences assigned to 7,759 unique protein groups (false discovery rate <1% on peptide and protein level; requiring a minimum of one unique peptide per protein). H/L protein ratios (H/L protein ratios of label swap experiments were inverted) were log_2_ transformed and quantile normalized (Supplementary Fig. 3a–c). We restricted our initial analysis to protein groups quantified with at least three unique peptides in all experiments (*n*=3,352). Comparing averaged log_2_ H/L protein ratios of lenalidomide treated or DMSO control samples (Pearson's correlation coefficient of 0.99), we identified CSNK1A1 as the most strongly affected protein (Fig. 2b), which is in accordance with the pSILAC experiments in HEK293T. We next assessed the statistical significance of drug-induced changes to protein turnover, which is reflected in the difference between H/L protein ratios of DMSO and lenalidomide samples. We calculated intensity binned (10 bins of equal size) *P* values using the significance B approach^35^ (Fig. 2c), which reflects the commonly observed dependence of H/L protein variability on signal intensity. In this approach, we identified additional proteins to exhibit significantly altered H/L protein ratios, most notably ZFP91 (Fig. 2b,c and Supplementary Table 3). To exclude the possibility of the drug treatment altering RNA levels, we performed RNA-seq on the samples used for mass spectrometry. The resulting data were analysed both at the levels of exons and introns, which allows the detection of both transcriptional and post-transcriptional changes^37^. However, very few genes displayed a significant change in transcript levels, suggesting that transcriptional changes can still be neglected at the T16 time point (Fig. 2d). Overall, the experimental design significantly reduced the required machine time compared to multi time point experiments. Notably, CSNK1A1 and ZFP91 were both also identified by analysing forward and reverse experiments individually (two replicates each), which indicates that two replicates would be sufficient to generate a list of candidates for further validation (Supplementary Fig. 1b,c).

### Comparing changes in protein stability and abundance

**Figure 3:**
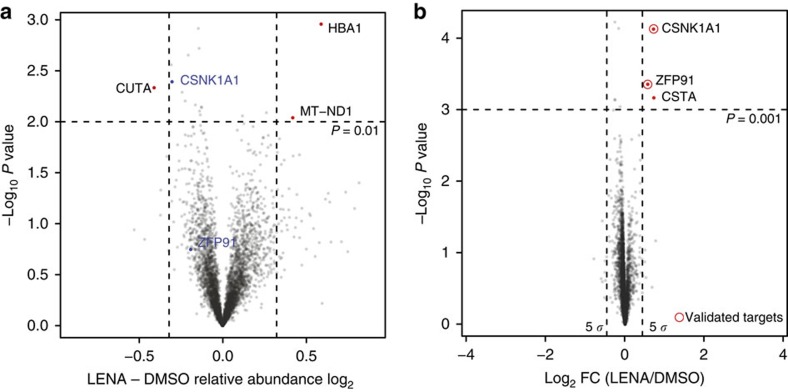
Combining multiple data sets can further increase robustness and sensitivity. (**a**) Scatter plot depicting the fold change in relative abundance comparing lenalidomide to DMSO control treatment (HEK293 TMT samples). Log_2_ fold changes are shown on the *x* axis and negative *P* values are shown on the *y* axis. The vertical dashed lines indicate ±0.32 log_2_ fold change (25% up- or downregulation) and the horizontal dashed line indicates *P* value <0.01 (*n*=2 biological replicates). (**b**) Linear model analysis across all forward samples (multi and single time point experiments) using limma (see Methods section) finds CSNK1A1, CSTA and ZFP91 as most significant hits (*P* value <0.001). The data are depicted as volcano plot showing differentially stable proteins following treatment with lenalidomide as compared with DMSO. The *x* axis shows the log_2_ fold change and the *y* axis refers to the associated *P* value (−log_10_ scale).

Next, we directly compared single time point pSILAC MS to measuring changes in total protein levels by TMT. We treated HEK293T cells for 16 h with 30 μM lenalidomide or a DMSO control (two biological replicates, consistent with the treatment for pSILAC, except that we did not exchange the growth medium for heavy amino acids). After lysis, reduction, alkylation and tryptic digest, peptides were subjected to TMT labelling, pre-fractionation and subsequent mass spectrometry analysis using a MS3 protocol on an Orbitrap Fusion mass spectrometer (see Methods section)^38^. Following scaling and normalization of reporter ion intensities, we performed statistical analysis by calculating moderated *t*-test *P* values using the limma package^36^ (Fig. 3a). While the two validated substrates CSNK1A1 and ZFP91 show induced degradation by lenalidomide, the observation, however, is not significant and neither CSNK1A1 nor ZFP91 were reliably identified as a drug target based on the TMT data set. In contrast, within the pSILAC HEK293T T16 samples as well as in the Hct116 T16 samples (see Figs 1g and 2b,c and Supplementary Fig. 1b,c), we unambiguously identify CSNK1A1 and ZFP91.

We conclude that for the target identification of small molecules that exert their effects by altering protein stability, measuring protein turnover provides a sensitive and robust alternative compared to relative quantification of changes to protein abundance.

### Combining multiple data sets can further increase robustness

We next explored the potential for combining data from multiple data sets to enhance the robustness of target identification. To do so, we utilized a linear model approach as implemented in the limma package^36^ and frequently employed for the analysis of gene expression data (see Methods section; and Supplementary Table 1 for design matrix). This framework allows modelling of both, the general changes to log_2_ H/L protein ratios over time and the influence of the drug treatment on these changes. When applied to H/L protein ratios of all forward pSILAC experiments (proteins with unique peptides <3 or missing values were excluded, resulting in 3,535 proteins analysed), we find CSNK1A1 and ZFP91 as the two most significant proteins (*P*<0.0005 and log-fold change >±0.5), with log_2_ fold changes of 0.72 and 0.57, respectively (Fig. 3b, Table 1 and Supplementary Table 4). Therefore, this approach is suitable to combine data from biological replicates and together with the previous results, prompted us to further validate ZFP91 as a novel lenalidomide-induced target of CRL4^CRBN^.

### ZFP91 is a lenalidomide-dependent CRL4^CRBN^ substrate

**Figure 4:**
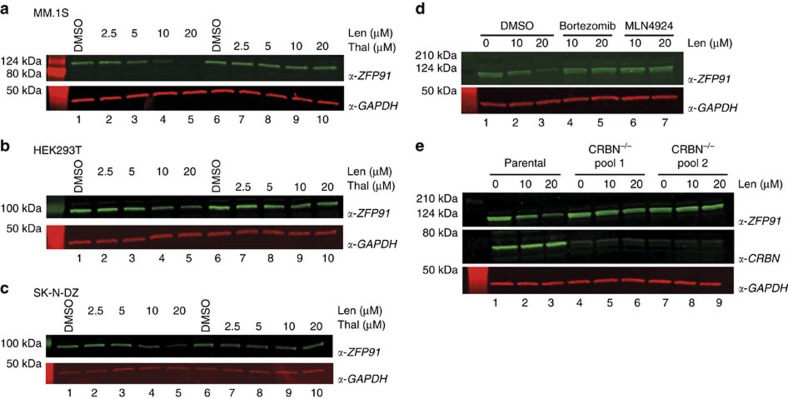
Validation of ZFP91 as *bona fide* lenalidomide-induced CRL4^CRBN^ substrate. (**a**) MM.1S cells were treated with increasing concentrations of lenalidomide or with DMSO. Following 24 h of incubation, ZFP91 and GAPDH levels were detected by anti-ZFP91 and anti-GAPDH western blot (shown is one representative experiment out of three replicates). (**b**) HEK293T cells were treated with 50μg ml^−1^ cycloheximide and increasing concentrations of lenalidomide, thalidomide or with DMSO, and cells were incubated for 6 h. ZFP91 and GAPDH levels were detected using anti-ZFP91 or anti-GAPDH immunoblotting (shown is one representative experiment out of five replicates). (**c**) as in (**b**) but using SK-N-DZ cells instead (shown is one representative experiment out of three replicates). (**d**) as in (**b**) but with co-treatment of bortezomib (proteasome inhibitor, lanes 4, 5) or MLN4924 (inhibitor of the NEDD8-activating enzyme, lanes 6,7). Shown is one representative experiment out of three replicates. (**e**) as in (**b**) using parental HEK293T (lane 1–3) or two independent pools of HEK293T cells with genetic inactivation of CRBN by CRISPR/Cas9 (shown is one representative experiment out of two replicates).

ZFP91 is a ZnF protein and putative ubiquitin ligase^31^,^32^. To recapitulate our mass spectrometry results, we treated MM.1S cells with increasing concentrations of lenalidomide, thalidomide or with DMSO control and performed western blot analysis. We found a dose-dependent decrease of ZFP91 protein when treated with lenalidomide (Fig. 4a). We next examined the effect of lenalidomide on ZFP91 half-life in cells, by performing CHX chase experiments in HEK293T and SK-N-DZ cells (SK-N-DZ were selected for higher levels of CRBN^9^ and to validate the results in a cell line different from the cell lines used for mass spectrometry experiments). Following treatment with 50 μg ml^−1^ CHX and increasing concentrations of lenalidomide or thalidomide, we found that ZFP91 protein levels were largely depleted after 6 h of treatment with lenalidomide in a dose-dependent manner (Fig. 4b,c). Greater depletion was observed in SK-N-DZ cells, in accordance with the higher levels of CRL4^CRBN^ ligase. Thalidomide in contrast had very little effect on the stability of ZFP91, similar to what has been previously observed for the ZnF proteins IKZF1 and IKZF3 (refs 13, 14, 39). We next confirmed that lenalidomide-dependent degradation of ZFP91 was abrogated by treatment with the proteasome inhibitor bortezomib (Fig. 4d, lanes 4–5) and the NEDD8-activating enzyme inhibitor MLN4924 (Fig. 4d, lanes 6–7), in line with a Cullin RING ligase and proteasome-dependent mechanism. To confirm the dependence of ZFP91 degradation on CRL4^CRBN^, we utilized CRISPR/Cas9 genome editing to generate two independent pools of HEK293T cells with genetic inactivation of CRBN. Treatment of these cells did not result in altered stability of ZFP91, which in contrast was readily degraded in parental HEK293T cells (Fig. 4e).

**Figure 5:**
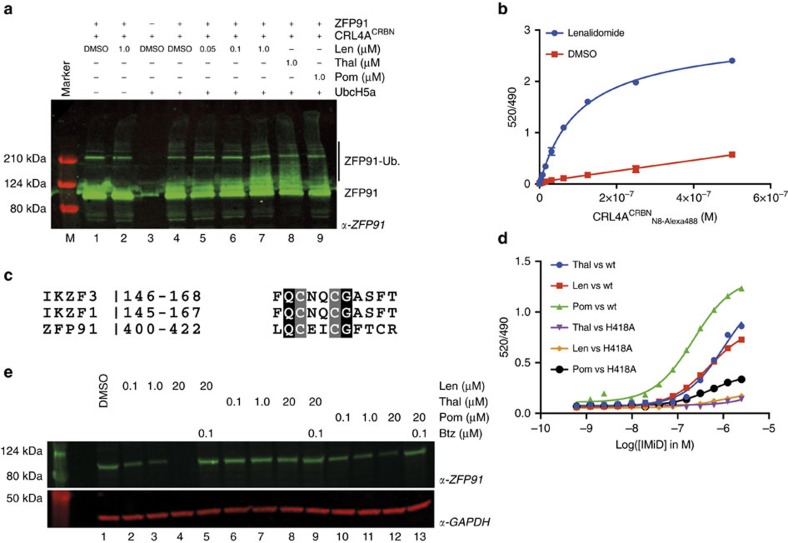
ZFP91 and IKZF1/3 share a common sequence motif. (**a**) *In vitro* ubiquitination of recombinant ZFP91 by recombinant _N8_CRL4A^CRBN^ is facilitated by lenalidomide (lanes 5–7), thalidomide (lane 8) and pomalidomide (lane 9). Shown is one representative experiment out of two replicates. (**b**) _Alexa488-N8_CRL4A^CRBN^ titrated to biotinylated wild-type ZFP91 at 100 nM in the presence of lenalidomide or DMSO as a control in presence of tracer Tb-streptavidin at 2 nM. Data are presented as means±s.d. (*n*=3). (**c**) Multiple sequence alignment of the putative ZFP91, IKZF1 and IKZF3 degron motifs^11^. Identical amino acids, and structural residues of the ZnF motif, are highlighted in black and grey, respectively. (**d**) Titration of thalidomide, lenalidomide and pomalidomide to _Alexa488-N8_CRL4A^CRBN^ at 0.2 μM, biotin-ZFP91 at 0.1 μM and Tb-streptavidin at 2 nM. EC50 values are shown and indicate preference for pomalidomide *in vitro*. Data are presented as individual data points for one representative experiment out of four replicates. (**e**) MM.1S cells were treated with increasing concentrations of lenalidomide, thalidomide, pomalidomide or a DMSO control for 12 h. Co-treatment with the proteasome inhibitor bortezomib was included as an additional control. ZFP91 and GAPDH levels were detected using anti-ZFP91 or anti-GAPDH immunoblotting (shown is the data for one representative experiment).

We next examined if CRL4A^CRBN^ binds to and ubiquitinates ZFP91 in a fully recombinant system. We subjected insect cell expressed and purified ZFP91 to *in vitro* ubiquitination by purified recombinant CRL4A^CRBN^ and found that in accordance with the cellular data, presence of lenalidomide promotes ubiquitination of ZFP91 in a dose-dependent manner (Fig. 5a, lanes 1–7). We further found that the close derivatives thalidomide and pomalidomide also promote the ubiquitination of ZFP91 *in vitro.*

This prompted us to characterize the binding of ZFP91 to CRL4A^CRBN^ *in vitro*. We measured the affinity of biotinylated ZFP91 to fluorescently labelled CRL4A^CRBN^ using time-resolved fluorescence energy transfer (TR-FRET)^11^. In presence of saturating concentrations of lenalidomide, we confirmed a tight association of ZFP91 with CRL4A^CRBN^ with an apparent dissociation constant (*K*_D_^app^) of 107±4 nM (Fig. 5b and Methods section). However, no binding was observed in the absence of compound. We had previously shown that CSNK1A1 and IKZF1/3 bind CRBN through a β-hairpin loop^11^, a structural feature also observed in GSPT1, the CRL4^CRBN^ substrate dependent on the IMiD analogue CC885 (ref. 40). Similar to IKZF1/3, ZFP91 contains ZnF domains, and sequence comparison reveals that ZnF4 of ZFP91 (residues 400–422) shares a key glycine with ZnF2 of IKZF1 (Fig. 5c). To test if ZnF4 is responsible for binding to CRL4^CRBN^, we mutated ZFP91 residue histidine 418 (one of the zinc-coordinating residues) to alanine (H418A), to disrupt the structural integrity of the ZnF4 domain. To test the effect on binding, we compared IMiD-induced binding of ZFP91 wild type and H418A mutant to CRL4A^CRBN^. While we observe dose-dependent binding of wild-type ZFP91 to CRL4A^CRBN^, the H418A mutation largely disrupts binding of ZFP91 to CRL4^CRBN^ (Fig. 5b,d). In accordance with the similarity to IKZF1/3, we found that thalidomide, lenalidomide and pomalidomide promote binding of ZFP91 to CRL4A^CRBN^ *in vitro*, however, the potency of the compounds relative to each other slightly differs from what was observed for IKZF1/3 (ref. 11). Following this observation, we next sought to test if pomalidomide and thalidomide would also promote ZFP91 degradation in cells. We treated MM.1S cells with increasing concentrations of thalidomide, lenalidomide and pomalidomide and found that lenalidomide and pomalidomide promote ZFP91 degradation to a similar extent, while the effect of thalidomide is minimal (Fig. 5e). This is in accordance with what has been reported for IKZF1/3 and could be a result of thalidomide being significantly less stable in cell culture medium than lenalidomide and pomalidomide^9^,^13^,^14^. We conclude that ZFP91 represents a *bona fide* lenalidomide-induced substrate for CRL4^CRBN^. Based on recent structural studies of the CRBN–lenalidomide–CSNK1A1 and CRBN–CC885–GSPT1 complexes^11^,^41^, we propose that a common sequence motif exists for IMiD-dependent binding of ZnF containing substrates to CRL4^CRBN^ (Fig. 5c).

## Discussion

The increasing number of small molecules directed at altering the stability and degradation of protein targets^20^,^21^,^22^,^23^,^42^, and natural products with potential activity towards protein stability^43^, require large-scale and proteome-wide methods to interrogate global on- and off-target effects. Here we introduce a novel approach to detect global changes to protein turnover in a single time point pSILAC mass spectrometry-based proteomics experiment. While it should be noted that single time point pSILAC does not report precise protein half-lives, and differences to H/L ratios can be the product of multiple effects (such as induced protein degradation/stabilization, altered translation or transcriptional effects); we demonstrate that the combination of short drug treatment and RNA-seq can largely mitigate these potential sources of false positives. The combination of pSILAC with TMT^44^ could further reduce the required machine time by combining replicates and multiple conditions into a single mass spectrometry experiment. However, such a strategy would likely come at the cost of sequencing depth due to necessary fragmentation of both (heavy and light) SILAC pairs to achieve TMT quantification. An alternative approach for a higher level of multiplexing could be neutron-encoded mass signatures SILAC (NeuCode-SILAC)^45^, which similarly to conventional SILAC can be applied in a pulsed manner.

By applying pSILAC to the potent anti-cancer therapeutic lenalidomide, we demonstrate that a single time point experiment could identify ZFP91 as novel lenalidomide-dependent CRL4^CRBN^ substrate. ZFP91 and the known target CSNK1A1 were consistently detected as *neo*-substrate candidates in all single time point pSILAC experiments. While calculated protein half-lives from the multi time point experiment robustly identified CSNK1A1, ZFP91 was dropped from half-life calculation due to missing values at one of the time points. Characterization of the ZFP91 interaction with CRL4^CRBN^ shows that a common structural motif in the ZnF4 and ZnF2 domains of ZFP91 and IKZF1/3, respectively, mediates a high-affinity interaction with a CRL4^CRBN^-IMiD complex. The plethora of ZnF proteins in the human proteome, together with the tolerance for amino-acid variation increases the probability that even more targets of IMiD-induced and CRL4^CRBN^-mediated degradation exist.

## Methods

### Compounds, enzymes and antibodies

Thalidomide (HY-14658), lenalidomide (HY-A0003), pomalidomide (HY-10984), MLN4924 (HY-70062) and bortezomib (HY-10227) were purchased from MedChem Express (MCE, USA). All compounds were dissolved in DMSO at various concentrations. Due to limited stability of thalidomide in aqueous solutions^9^, all required dilutions and cell culture media containing compounds were prepared fresh. HEK293T, SK-N-DZ, MM.1S and Hct116 cell lines were purchased from ATCC and cultured according to ATCC instructions. Cell lines were routinely tested for mycoplasma contamination using the MycoAlert detection kit (Lonza). Arginine and lysine-free cell culture medium, dialysed foetal bovine serum and amino acids for pulse-SILAC mass spectrometry were purchased from Cambridge isotope (Cambridge isotope laboratories, USA). Proteins were tested for >99% SILAC amino-acid incorporation after six–ten passages by mass spectrometry. Sequencing grade modified trypsin (V5117) was purchased from Promega (Promega, USA) and mass spectrometry grade lysyl endopeptidase from Wako (Wako Pure Chemicals, Japan). Primary and secondary antibodies used included anti-ZFP91 at 1:100–1:500 dilution (ab30970, abcam), anti-ZFP91 at 1:250–1:5,000 dilution (A303-245A, Bethyl Laboratories), anti-CRBN at 1:250 dilution (NBP1–91810, Novus), anti-GAPDH at 1:10,000 dilution (G8795, Sigma), IRDye680 Donkey anti-mouse at 1:10,000 dilution (926–68072, LiCor) and IRDye800 Goat anti-rabbit at 1:10,000 dilution (926–32211, LiCor). Ubiquitination enzymes Uba1, UbcH5a and ubiquitin were purchased from Boston Biochem.

### Cell culture

HEK293T and Hct116 cells were cultured in L-arginine and L-lysine-free DMEM supplemented with dialysed foetal bovine serum, 2 mM L-glutamine and unlabelled L-arginine and L-lysine. Cells were grown to 40–50% confluency and the medium exchanged for DMEM supplemented with heavy L-arginine (13C6, 15N4—R10) and heavy L-lysine (13C6, 15N2—K8) to obtain heavy SILAC medium. Simultaneously, the medium was either supplemented with 30 μM lenalidomide (100 mM solution in DMSO) or the equivalent amounts of DMSO as control. Cells were incubated at 37 °C, 5% CO_2_ and collected at various time points by two washes with phosphate-buffered saline and direct addition of lysis buffer. If samples were split for parallel RNA-seq, cells were washed twice with phosphate-buffered saline, (Gibco) and split into two halves before lysis or RNA extraction. Total RNA was extracted using the RNeasy Mini Kit (Qiagen) following the manufacturer's instructions.

### Constructs and protein purification

Human DDB1, human CRBN, human CUL4A and mouse RBX1 were subcloned into pAC-derived vectors^46^ and recombinant proteins expressed as N-terminal His6 fusion proteins in *Trichoplusia ni* High Five insect cells using the baculovirus expression system (Invitrogen)^9^. Recombinant CRL4A^CRBN^ complex was purified through sequential Ni-NTA affinity, anion exchange (poros 50HQ) and size exclusion (Superdex 200) chromatography. *In vitro* neddylation was carried out as described^9^,^11^, CRL4A^CRBN^ was incubated with a reaction mixture containing Alexa488-NEDD8(M1C) at 3.8 μM, NAE/UBA1 (E1) at 50 nM, and UBC12 (E2), 1 mM ATP, 50 mM Tris pH 7.5, 100 mM NaCl, 2.5 mM MgCl_2_, 0.5 mM DTT and 5% (v/v) glycerol for 2 h at room temperature. Neddylated CRL4A^CRBN^ was purified by size exclusion chromatography. Full-length human ZFP91 was obtained as synthetic codon optimized gBlock from IDT (IDT, USA) and cloned into pAC-derived vectors^46^. Mutant ZFP91 H418A was derived from this construct by Q5 mutagenesis (NEB, USA). Recombinant ZFP91 was expressed as N-terminal StrepII fusion protein in *Trichoplusia ni* High Five insect cells using the baculovirus expression system (Invitrogen). For purification of StrepII-ZFP91, cells were lysed by sonication in lysis buffer (50 mM Tris-HCl pH 8.0, 200 mM NaCl, 5 mM 2-mercaptoethanol (BME), 1 mM phenylmethylsulfonyl fluoride, 0.1% Triton X-100 and 1 × protease inhibitor cocktail (Roche). Following ultracentrifugation, the soluble fraction was passed over Strep-Tactin Sepharose (IBA) and eluted with 2.5 mM D-desthiobiotin in wash buffer (50 mM Tris-HCl, 200 mM NaCl, 5 mM BME). The affinity purified protein was diluted to 50 mM NaCl with dilution buffer (50 mM Tris-HCl pH 8.5, 0.5 mM tris(2-carboxyethyl)phosphine (TCEP)) and further purified via anion exchange chromatography (Poros 50HQ) with a linear gradient of 0–80% buffer B (50 mM Tris-HCl pH 8.5, 1 M NaCl, 0.5 mM TCEP) in buffer A (50 mM Tris-HCl pH 8.5, 25 mM NaCl, 0.5 mM TCEP) and subjected to size exclusion chromatography in 50 mM HEPES pH 7.4, 200 mM NaCl and 0.5 mM TCEP. The protein containing fractions were pooled, concentrated using ultrafiltration (Millipore) and flash frozen in liquid N_2_. Proteins were stored at −80 °C.

### Biotinylation of ZFP91

Purified StrepII-Avi-tagged wild-type or mutant ZFP91 were biotinylated *in vitro* at a concentration of 5–20 μM by incubation with final concentrations of 2.5 μM BirA enzyme and 0.2 mM D-biotin in 50 mM HEPES pH 7.4, 200 mM NaCl, 10 mM MgCl_2_, 0.25 mM TCEP and 20 mM ATP. The reaction was incubated for 1 h at room temperature and stored at 4 °C for 14–16 h. Biotinylated proteins were purified by gel filtration chromatography and stored at −80 °C.

### TR-FRET binding assay

Increasing concentrations of Alexa488-NEDD8-labelled CRL4^CRBN^ (_N8_CRL4^CRBN^)^47^ were added to pre-mixed biotinylated ZFP91 at 100 nM, terbium-coupled streptavidin at 2 nM (Invitrogen) and IMiDs at 5 μM (final concentrations) in 384-well microplates (Greiner, 784076) in a buffer containing 50 mM Tris pH 7.5, 100 mM NaCl, 0.1% pluronic acid and 1% DMSO. After excitation of terbium (Tb) fluorescence at 337 nm, emission at 490 nm (Tb) and 520 nm (Alexa488) were recorded with a 70 μs delay to reduce background fluorescence and the reaction was followed over 1 h by recording 60 technical replicates of each data point using a PHERAstar FS microplate reader (BMG Labtech). The TR-FRET signal of each data point was extracted by calculating the 520/490 nm ratio. Data were analysed with GraphPad Prism 6 assuming equimolar binding of the probe to the receptor using the following equation^11^,^47^:


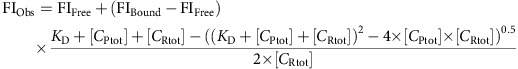


FI_Obs_ is the observed fluorescence intensity, and FI_Free_ and FI_Bound_ the fluorescence intensities of the probe in its free and its bound states, respectively.

Dose response curves for IMiDs were obtained by mixing increasing concentrations of IMiD with a reservoir containing _N8_CRL4A^CRBN^ at 200 nM, biotin-ZFP91 or biotin-ZFP91(H418A) at 100 nM, and Tb-Streptavidin at 2 nM. The fluorescence as 520/490 nm ratio were recorded over 1 h as 60 technical replicates of each data point using a PHERAstar FS microplate reader (BMG Labtech) and resulting data analysed with GraphPad Prism 6.

### *In vitro* ubiquitination assays

*In vitro* ubiquitination was performed by mixing ZFP91 at 0.6 μM, and _N8_CRL4^CRBN^ at 80 nM with a reaction mixture containing IMiDs at indicated concentrations or a DMSO control, E1 (UBA1, Boston Biochem) at 40 nM, E2 (UBCH5a, Boston Biochem) at 1.2 μM, ubiquitin at 23 μM. Reactions were carried out in 50 mM Tris pH 7.5, 30 mM NaCl, 5 mM MgCl_2_, 0.2 mM CaCl_2_, 2.5 mM ATP, 0.1% Triton X-100 and 0.1 mg ml^−1^ BSA, incubated for 15–30 min at 30 °C and analysed by western blot using anti-ZFP91 and anti-rabbit IRDye 800CW antibodies. Blots were scanned on a LICOR Odyssey infrared imaging system (uncropped immunoblots are provided in Supplementary Fig. 4).

### HPLC and mass spectrometry

The multi time point mass spectrometry experiment consisted of two replicates each for DMSO and LENA-treated cells collected at 6 h (T6), 10 h (T10.raw data files were initially mislabelled as T8) and 16 h (T16) post treatment. One additional reverse experiment (label swap) was conducted at the T6 and T10 time points. During data analysis, we noticed a dramatically reduced MS signal for the T10 forward samples, which we believe was due to reduced peptide yields from fractionation and digest (the reverse samples processed at a different time were unaffected). These samples were removed from all downstream analysis. Since we did not aim to generate precise protein half-life values, we refrained from repeating the experiments. For the multi time point experiment, cells were lysed in RIPA buffer (50 mM Tris pH 8.0, 150 mM NaCl, 0.1% SDS, 0.5% sodium deoxycholate, 1% Triton X-100, 1 tablet per 100 ml Sigma protease inhibitor cocktail) for 30 min on ice. 200 μg of lysate was subjected to reduction, alkylation and subsequent SDS–PAGE separation. SDS–PAGE gels were fixed in fixing solution (10% v/v acetic acid and 50% v/v methanol) for 30 min and whole gel lanes cut into 15 slices of equal size. Gel pieces were individually subjected to in-gel tryptic digest. Peptides were analysed by nano liquid chromatography tandem mass spectrometry with an EASY-nLC 1,000 pump using a two-column set-up (Thermo Scientific). The acidified peptides were loaded with buffer A onto a trap column (C_18_ Acclaim PepMap 100 trap column 75 μm × 2 cm, 3 μm 100 Å) at a flow rate of 200 nl min^−1^. They were separated with a linear gradient of 2–5% buffer B in 5 min followed by 5–30% B in 150 min, 30–50% B in 30 min, 50–80% B in 5 min and by 5 min wash at 80% buffer B (buffer A: 0.1% formic acid in water, buffer B: 0.1% formic acid in acetonitrile) on a 75 μm × 25 cm Reprosil-PUR C_18_, 3 μm, 100 Å PicoFrit column at 60 °C mounted on a DPV ion source (New Objective) connected to an Orbitrap VELOS mass spectrometer (Thermo Scientific). The data were acquired in a mass range of *m/z* 350–1,300, resolution 60,000, AGC 2 × 10^6^, maximum injection time 200 ms, dynamic exclusion 15 s for the peptide measurement in the Orbitrap, a top 20 method with CID fragmentation of the most abundant peptides at a maximum injection time 50 ms, AGC 1 × 10^4^, NCE 35% and fragment ion measurement in the LTQ.

For the single time point pulse-SILAC experiment (two replicates per condition forward and two replicates per condition reverse for a total of four technical replicates), cells were lysed in buffer containing 0.5 M Tris pH 8.6 and 6 M GnHCl. An aliquot of 200 μg of protein lysate was reduced in 16 mM TCEP for 30 min and alkylated in 35 mM iodoacetamide for 30 min in the dark. The proteins were digested at 37 °C with lysyl endopeptidase (Wako) after dilution to ≈2 M GnHCl (with 50 mM Tris-HCl pH 7.3, 5 mM CaCl_2_ buffer) for 6 h, and after dilution to <1 M GnHCl with trypsin (Promega) at 37 °C overnight. The resulting peptides were desalted using 50 mg C_18_ solid-phase extraction cartridges (Waters) and offline fractionated into 36 fractions by high pH reversed phase chromatography on an Agilent1100 system. Between 25 and 40 μg of peptides were loaded in high pH buffer A and separated at a flow rate of 12 μl min^−1^ with a gradient of 2–10% high pH buffer B in 10 min followed by a linear increase from 10–35% B in 60 min, 35–100% B in 5 min followed by 5 min wash at 100% B (high pH buffer A: 20 mM ammonium formate at pH 10, high pH buffer B: 20 mM ammonium formate at pH 10 in 90% acetonitrile) on a YMC-Triart C18 0.5 × 250 mm, 12 nm × 3 μm column. The 36 fractions were pooled (fr01+fr13+fr25, fr02+fr14+fr26 and so on) to a final of 12 samples.

Peptides were analysed by nano liquid chromatography tandem mass spectrometry with an EASY-nLC 1,000 pump using a two-column set-up (Thermo Fisher Scientific). The peptides were loaded in buffer A onto a trap column (Acclaim PepMap 100, 75 μm × 2 cm, C18, 3 μm, 100 Å). They were separated, at a flow rate of 150 nl min^−1^ with a linear gradient of 2–6% buffer B in 8 min followed by a linear increase from 6–22% B in 88 min, 22–28% B in 16 min, 28–36% B in 8 min, 36–80% B in 4 min followed by 10 min wash at 80% B (buffer A: 0.1% formic acid in water, buffer B: 0.1% formic acid in acetonitrile) on a PepMap RSLC analytical column (50 μm × 15 cm, C18, 2 μm) at 45 °C mounted on a modified DPV ion source (New Objective) connected to an Orbitrap Fusion mass spectrometer (Thermo Scientific). The data were acquired using a mass range of *m/z* 350–1,600, resolution 120,000, AGC target 3 × 10^5^, maximum injection time 100 ms, dynamic exclusion of 60 s for the peptide measurements in the Orbitrap and a cycle time of 3 s for HCD fragmentation of the most abundant peptides and fragment ion measurement in the LTQ using AGC target 1 × 10^2^, NCE 30%, maximum injection time 250 ms.

### Mass spectrometry data analysis

MaxQuant^35^ (versions 1.5.0.30 and 1.5.3.8 for multi time point and single time point, respectively) was used for RAW file processing and controlling peptide and protein level false discovery rates, assembling proteins from peptides, and protein quantification from peptides. Fragment ion spectra were searched against a human Uniprot database (downloaded on 13 November 2014 and 29 January 2015 for multi time point and single time point, respectively) and common contaminant proteins (included in the MaxQuant software). Carbamidomethylation (Cys) was set as a fixed modification; deamidation (Gln, Asn) and oxidation (Met) were selected as variable modifications. SILAC amino acids used for quantification were Lys8 and Arg10. The MaxQuant ProteinGroups.txt output file was imported into the R framework^48^ for all subsequent analyses.

Before statistical analysis proteins flagged as contaminants, reverse (decoy) proteins and proteins only identified by site were removed from the data set. H/L protein ratios (‘Ratio H/L' columns per time point, not normalized) were quantile normalized using the limma package^36^ with normalization carried out separately for each time point. The quantile normalization as described elsewhere^49^ and implemented in the limma package has the advantage of being a complete data method without requiring a defined baseline data set. In short, *n* data sets of length *p* are turned into a matrix *X* with *p* × *n* dimensions, with each data set being a column. We sort each column of *X* to give *X*_sort_ and now assign the mean across each row of *X*_sort_ to each element of the row to give *X'*_Sort_. Next, we rearrange each column of *X'*_Sort_ back to the original ordering and derive *X*_Normalised_.

### Determination of protein half-lives

While we performed the linear regression fits to obtain protein half-lives, we noted several challenges for accurate determination of protein half-lives, including low number of time points, low number of replicates, inclusion of label swap data (which display a consistent rank order but a systematic offset compared to the forward experiments, see also Supplementary Fig. 3), and also the known limitations of obtaining half-lives by SILAC mass spectrometry, such as ‘light' amino-acid recycling^27^. However, we demonstrate that despite these limitations the data are informative for the identification of ligase targets. Protein half-lives were obtained as previously described^24^. In essence, normalized and log_2_ transformed H/L ratios for protein groups quantified across all time points and replicates (*n*=2,837) were used for linear regression (log_2_(H/L) ∼time) for the DMSO and lenalidomide samples of each protein. Proteins that exhibited sufficient quality of both (DMSO and lenalidomide) fits (*r*^2^>0.9, total number of proteins 2,759) were used to obtain the protein decay rate constant (*k*_dp_) using the following equation^24^:


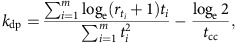


where *m* is the number of time points (*t*_*i*_), *r*_*t_i_*_ the H/L ratio of a specific protein at a given time point, and *t*_cc_ is the time needed to double the total amount of protein in the experiment, which is usually set to the duration of the cell cycle. The doubling time of HEK293T cells was estimated to be ≈22 h by counting cells growing on 100 mm dishes. However, using *t*_cc_=22 h resulted in negative decay rates for a subset of proteins, indicating that in our experimental set-up the protein doubling time was longer than 22 h. We attributed this effect to the cells growing more slowly than expected during the experiment, for example, due to increasing cell densities or the effect of heavy amino acids. To accommodate for this effect, *t*_cc_ was set to 100 h, which resulted in a mean protein half-life of 44.6 h similar to published estimates^24^. While the use of an incorrect *t*_cc_ value leads to incorrect absolute protein decay rates, it does not limit the use of the inferred decay rates or protein half-lives as a relative measure in the comparison of different treatment conditions, which is the purpose in this study. Based on the decay constant, the half-life of a protein is given by^24^:


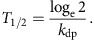


The derived protein half-lives were used for subsequent comparison.

### Data analysis for single time point pulse SILAC

For conventional SILAC mass spectrometry, it has been shown that the distance of a ratio *r* measured in terms of the s.d. can serve as a robust estimate of its significance. Under the assumption of a normal distribution of the data, a *P* value can be calculated (significance A)^35^. Based on the observation that the error of H/L protein ratios is correlated to the summed intensity, *P* values are calculated for subsets of the data binned by intensity (significance B)^35^. Since the log-transformed LENA/DMSO protein ratios assume in approximation a normal distribution, we utilized this approach to assess the statistical significance of proteins.

To control for potential non-linearities in some samples, log_2_ H/L protein ratios were quantile normalized using the normaliseBetweenArrays function from the limma package^36^ (see description of quantile normalization above). Protein ratios were filtered to include only proteins quantified by at least three unique peptides (not razor) and with a minimum of two peptides (unique and razor) in every individual LC-MS/MS experiment (no missing values for protein ratios). The remaining 3,352 protein ratios were used for subsequent analysis: median of DMSO and median of lenalidomide-treated H/L protein ratios were used to calculate LENA/DMSO protein ratios (ΔH/L). The ΔH/L ratios were binned by summed intensities (10 bins of equal size) and the 15.87, 50 and 84.13 percentiles *r*_−1_, *r*_0_ and *r*_1_ were calculated for each of the bins. For each protein, a significance B *P* value^35^ was calculated with the *r*_−1_, *r*_0_ and *r*_1_ values corresponding to their intensity bin and by using the following formula:


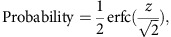


with the error function erfc defined as: 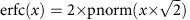, where pnorm() is the *R* function for cumulative distribution function for the normal distribution. Further *z* is defined as the distance measured in terms of the s.d.:


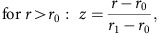



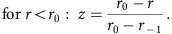


A list of all significance B values is provided (see Supplementary Table 3). A *P* value <1 × 10^−7^ was selected as cutoff.

### MS analysis across experiments using linear models

Log_2_ transformed H/L protein ratios were analysed using the limma package^36^ (see Supplementary Table 1 for design matrix). Intensities in mass spectrometry experiments have been found to correlate with the quality of quantification, log_10_ transformed intensities were therefore included as weighting factor for the linear model analysis. A list of log_2_ fold changes and statistical information for all protein groups is provided (see Supplementary Table 4).

### Sample preparation TMT LC-MS3 mass spectrometry

Samples were prepared as previously described^50^ with the following modification. All solutions are reported as final concentrations. Lysis buffer (8 M Urea, 1% SDS, 50 mM Tris pH 8.5, protease and phosphatase inhibitors from Roche) was added to the cell pellets to achieve a cell lysate with a protein concentration between 2 and 8 mg ml^−1^. A micro-BCA assay (Pierce) was used to determine the final protein concentration in the cell lysate. Proteins were reduced and alkylated as previously described. Proteins were precipitated using methanol/chloroform. In brief, four volumes of methanol was added to the cell lysate, followed by one volume of chloroform and finally three volumes of water. The mixture was vortexed and centrifuged to separate the chloroform phase from the aqueous phase. The precipitated protein was washed with one volume of ice-cold methanol. The washed precipitated protein was allowed to air dry. Precipitated protein was resuspended in 4 M urea, 50 mM Tris pH 8.5. Proteins were first digested with LysC (1:50; enzyme:protein) for 12 h at 25 °C. The LysC digestion was diluted down to 1 M urea, 50 mM Tris pH 8.5 and then digested with trypsin (1:100; enzyme:protein) for another 8 h at 25 °C. Peptides were desalted using a C_18_ solid-phase extraction cartridges as previously described. Dried peptides were resuspended in 200 mM EPPS, pH 8.0. Peptide quantification was performed using the micro-BCA assay (Pierce). The same amount of peptide from each condition was labelled with TMT reagent (1:4; peptide:TMT label) (Pierce). The 10-plex labelling reactions were performed for 2 h at 25 °C. Modification of tyrosine residue with TMT was reversed by the addition of 5% hydroxyl amine for 15 min at 25 °C. The reaction was quenched with 0.5% TFA and samples were combined at a 1:1:1:1:1:1:1:1:1:1 ratio. Combined samples were desalted and offline fractionated into 24 fractions as previously described.

### LC-MS3 for TMT relative quantification

Twelve of the 24 peptide fraction from the high pH reverse phase step (every other fraction) were analysed with an LC-MS3 data collection strategy^38^ on an Orbitrap Fusion mass spectrometer (Thermo Fisher Scientific) equipped with a Proxeon Easy-nLC 1,000 for online sample handling and peptide separations. Approximately 5 μg of peptide resuspended in 5% formic acid+5% acetonitrile was loaded onto a 100 μm inner diameter fused-silica micro capillary with a needle tip pulled to an internal diameter less than 5 μm. The column was packed in-house to a length of 35 cm with a C_18_ reverse phase resin (GP118 resin 1.8 μm, 120 Å, Sepax Technologies). The peptides were separated using a 180 min linear gradient from 3 to 25% buffer B (100% acetonitrile+0.125% formic acid) equilibrated with buffer A (3% acetonitrile+0.125% formic acid) at a flow rate of 400 nl min^−1^ across the column. The scan sequence for the Fusion Orbitrap began with an MS1 spectrum (Orbitrap analysis, resolution 120,000, 400–14,000 *m/z* scan range with quadrupole isolation, AGC target 2 × 10^5^, maximum injection time 100 ms, dynamic exclusion of 60 s). ‘Top N' (the top 10 precursors) was selected for MS2 analysis, which consisted of CID ion trap analysis (AGC 8 × 10^3^, NCE 35, maximum injection time 150 ms), and quadrupole isolation of 0.5 Da for the MS1 scan. The top 10 fragment ion precursors from each MS2 scan were selected for MS3 analysis (synchronous precursor selection), in which precursors were fragmented by HCD prior to Orbitrap analysis (NCE 55, max AGC 1 × 10^5^, maximum injection time 150 ms; MS2 quadrapole isolation was set to 2.5 Da, resolution 60,000).

### LC-MS3 data analysis

A suite of in-house software tools (Thermo Center for Multiplexed Proteomics at Harvard Medical School) were used to for.RAW file processing and controlling peptide and protein level false discovery rates, assembling proteins from peptides, and protein quantification from peptides as previously described. MS/MS spectra were searched against a Uniprot human database (February 2014) with both the forward and reverse sequences. Database search criteria are as follows: tryptic with two missed cleavages, a precursor mass tolerance of 50 ppm, fragment ion mass tolerance of 1.0 Da, static alkylation of cysteine (57.02146 Da), static TMT labelling of lysine residues and N-termini of peptides (229.16293 Da) and variable oxidation of methionine (15.99491 Da). TMT reporter ion intensities were measured using a 0.003 Da window around the theoretical *m/z* for each reporter ion in the MS3 scan. Peptide spectral matches with poor quality MS3 spectra were excluded from quantification (<summed signal-to-noise across 10 channels and <0.5 precursor isolation specificity).

Reporter ion intensities were normalized and scaled in the R framework^48^. Statistical analysis was carried out using the limma package within the R framework^36^.

### RNA-seq analysis

Samples of isolated RNA were used for library preparation using ScriptSeq v2 total RNA library preparation kit and sequenced on an Illumina HiSeq 2,500 sequencer per Illumina standards. Sequencing reads were aligned to the human genome (Bsgenome.Hsapiens.UCSC.hg19 Bioconductor package, using splicedAlignment=TRUE) and quantified at the level of genes (TxDb.Hsapiens.UCSC.hg19.knownGene Bioconductor package) using the QuasR package with default parameters^51^. Intronic and exonic reads were separately quantified and differentially expressed genes were identified using the edgeR Bioconductor package^52^ as described elsewhere^37^.

### Data availability

All RNA-seq data sets have been deposited in GEO under the accession number GSE94728. Mass spectrometry raw data files have been deposited in PRIDE Archive under the accession numbers PXD005857 and PXD005849 for the HEK293T and Hct116 experiments, respectively. The data that support the findings of this study are available from the corresponding author on request.

## Additional information

**How to cite this article:** An, J. *et al*. pSILAC mass spectrometry reveals ZFP91 as IMiD-dependent substrate of the CRL4^CRBN^ ubiquitin ligase. *Nat. Commun.* **8,** 15398 doi: 10.1038/ncomms15398 (2017).

**Publisher's note:** Springer Nature remains neutral with regard to jurisdictional claims in published maps and institutional affiliations.

## Supplementary Material

Supplementary Information Supplementary Figures and Supplementary Table

Supplementary Data 1 Limma output for single time point pSILAC in HEK293T cells.

Supplementary Data 2 Statistical analysis of single time point pSILAC in Hct116 cells.

Supplementary Data 3 Limma output for analysis of across multiple experiments.

## Figures and Tables

**Table 1:** List of proteins with altered stability.

**Significance B analysis single time point**			**Limma analysis across all samples**		
**Gene ID**	**LogFC**	***P*** **value**	**LogFC**	***P*** **value**	**ΔHalf-life**
Down					
CSNK1A1	0.62	2.2 × 10^−85^	0.72	7.6 × 10^−5^	Hit
CA2	0.29	4.1 × 10^−15^	0.08	0.07	No hit
LASP1	0.13	5.3 × 10^−10^	0.05	0.51	No hit
ZFP91	0.34	7.9 × 10^−8^	0.57	0.0004	No hit*
Up					
NAA30	−0.40	3.0 × 10^−22^	−0.18	0.39	No hit
NCSTN	−0.39	2.3 × 10^−21^	−0.32	0.07	No hit
POMP	−0.32	3.6 × 10^−14^	−0.12	0.34	No hit
GNL3L	−0.29	3.0 × 10^−12^	−0.41	0.06	No hit

Log_2_ fold changes are shown together with their respective *P* values. For half-life analysis, we qualitatively designated a protein a ‘hit' or ‘no hit'.

^*^ZFP91 was dropped from the half-life analysis for a missing value in one time point and is therefore designated ‘no hit'.
