# Supplementary material for: pSILAC mass spectrometry reveals ZFP91 as IMiD-dependent substrate of the CRL4CRBN ubiquitin ligase
Source: Nat Commun. 2017 May 22;8:15398. doi: 10.1038/ncomms15398 (PMC5458144; doi:10.1038/ncomms15398)
Supplement: Supplementary Information — Supplementary Figures and Supplementary Table [file ncomms15398-s1.pdf]

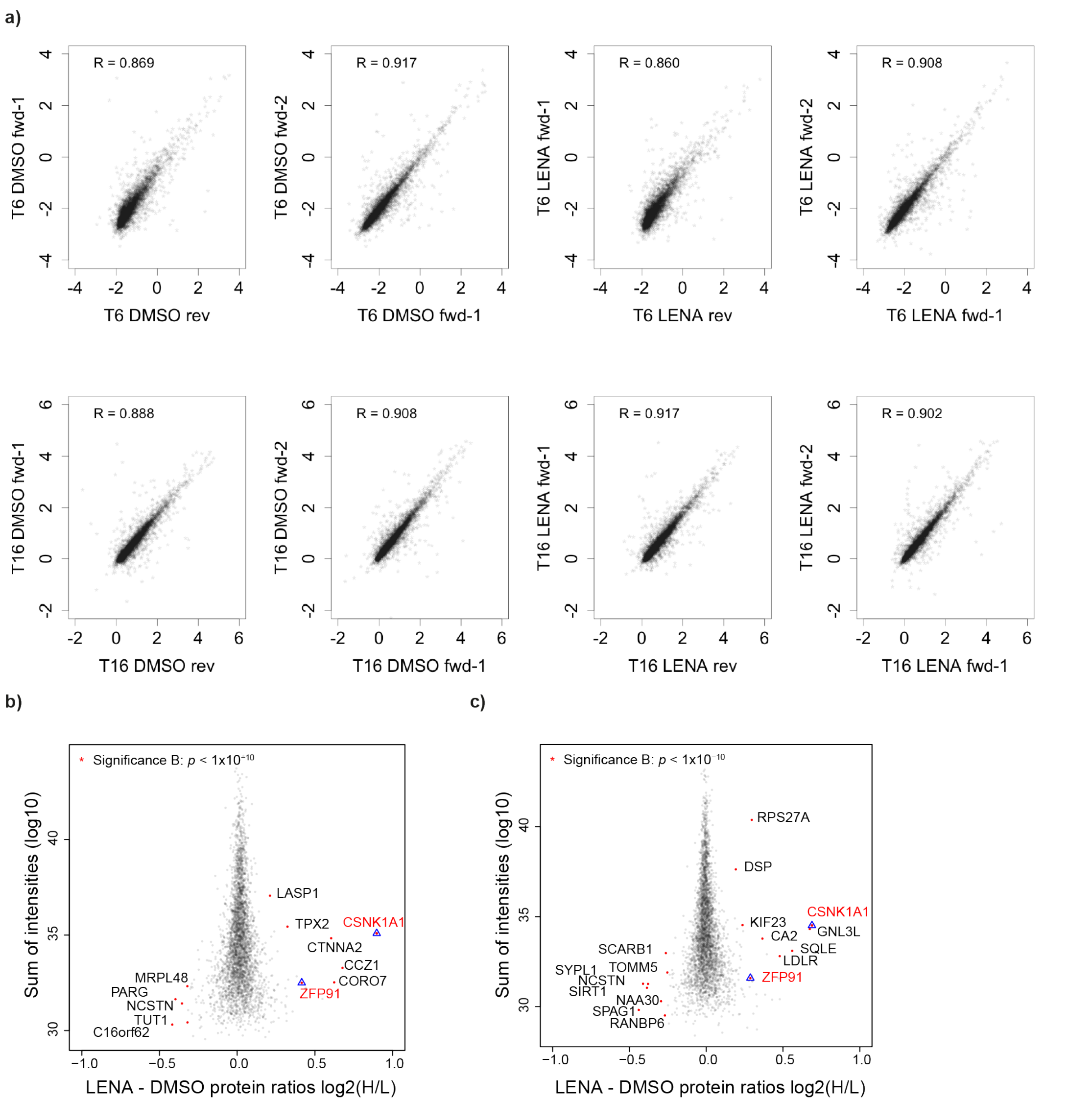

Supplementary Figure 1 | SILAC label effects do not negatively affect the experimental outcome.

a, Scatter plots comparing pairwise log2 H/L protein ratios of forward (light → heavy) and reverse (heavy → light, sign reversed) experiments or two replicate forward experiments. *R* indicates the Pearson's correlation coefficient. More pronounced label effects (offset from the diagonal indicating difference in growth behaviour) are observed for early time points. The overall correlation and the outcome of our pilot experiments suggest that the negative influence of SILAC does not prevent identification of changes to protein turnover. **b**, To exclude possible SILAC artifacts, forward and reverse samples (n=2 for each) of T16 Hct116 single time point pulse-SILAC experiments were analysed individually. Scatter plots depict the identification of substrate candidates in Hct116 T16 forward samples. Log2 changes in LENA to DMSO H/L protein ratio are shown on the x-axis, and log10 sum of MS1

intensities (combined for heavy and light peptides) on the y-axis. Significance *B* was calculated for 10 intensity bins and proteins with Significance $B < 1x10^{-10}$ are shown in red. Only protein groups that were quantified with minimum of 3 unique peptides in each experiment (3352) are shown. **c**, as in **b** but using only reverse experiments. The validated substrates, ZFP91 and CSNK1A1, were independently identified in forward and reverse experiments.

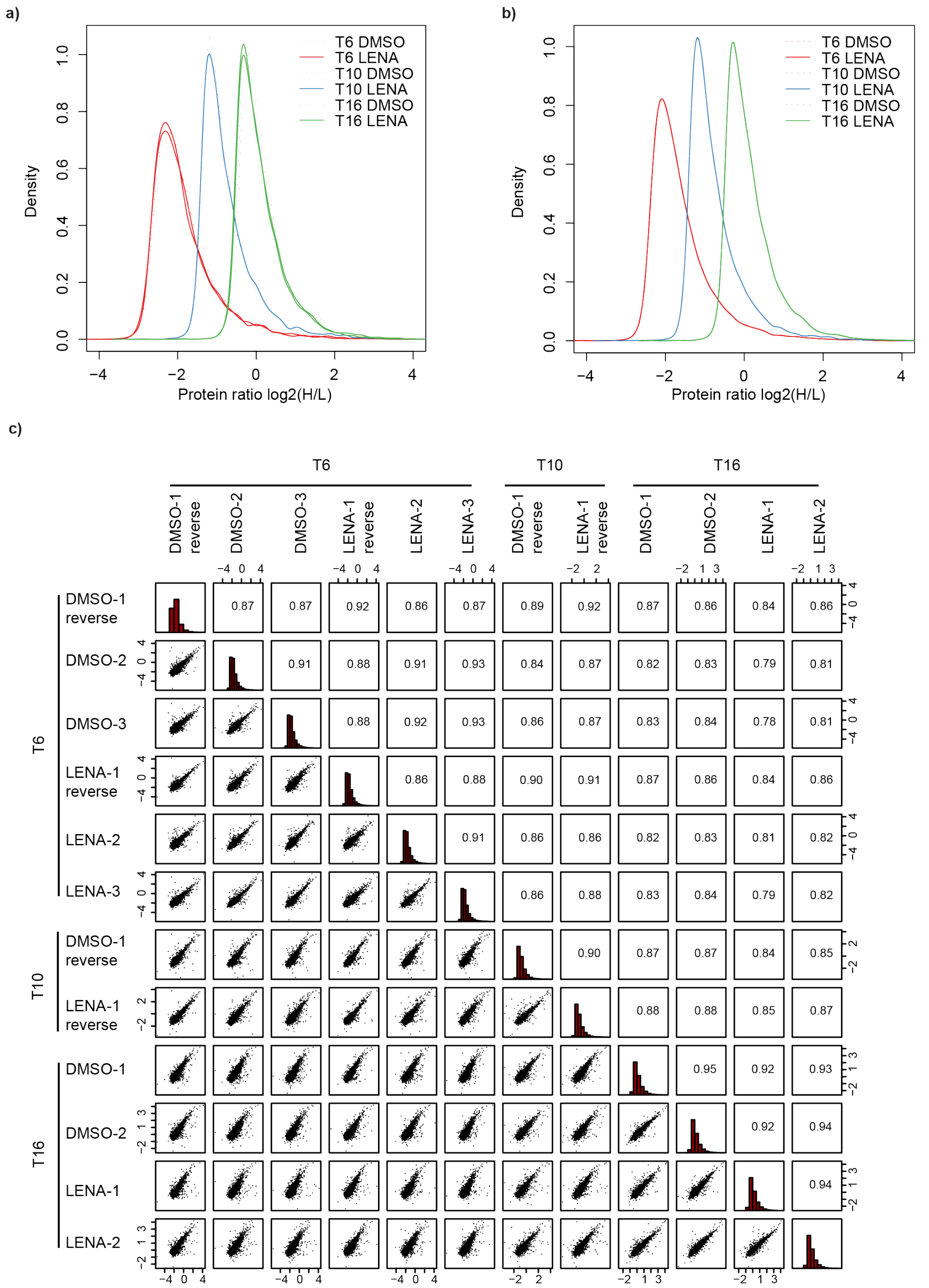

Supplementary Figure 2 | pulse-SILAC mass spectrometry in HEK293T cells.
a, Distribution of log2 H/L protein ratios before normalisation. **b**, Distribution of log2 H/L protein ratios after quantile normalisation. **c**, Pairwise Pearson's correlations coefficients (above diagonal), histograms (diagonal) and pairwise scatter plots (below

diagonal) of quantile normalised log2 H/L protein ratios for all samples of the multi time point pulse-SILAC experiment used for final analysis.

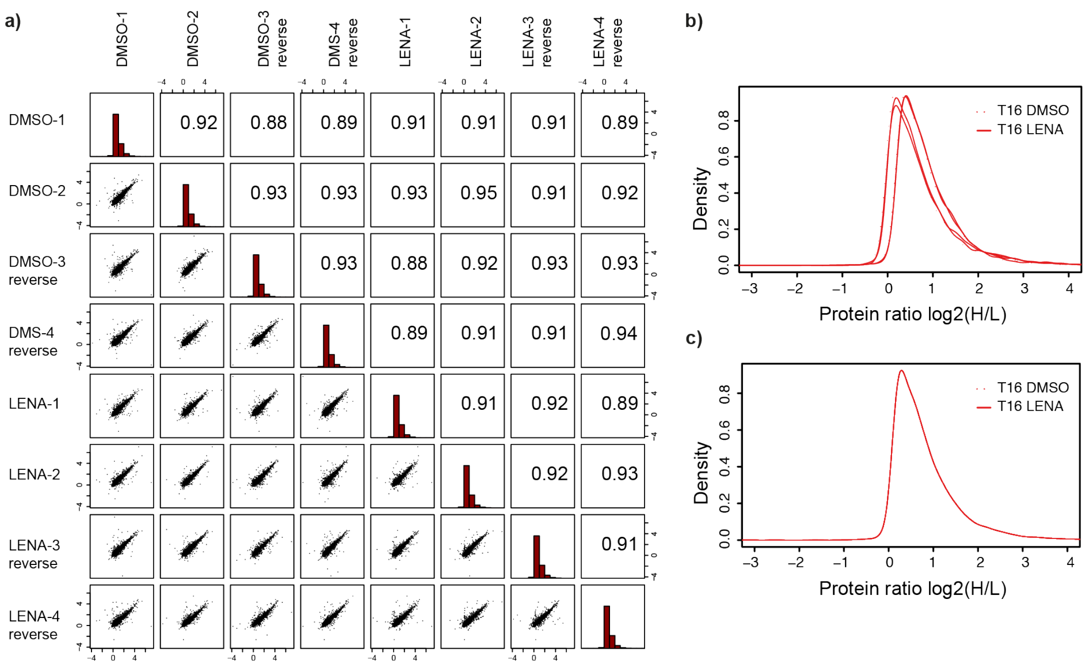

Supplementary Figure 3 | pulse-SILAC mass spectrometry in Hct116

a, Pairwise Pearson's correlation coefficients (above diagonal), histograms (diagonal) and pairwise scatter plots (below diagonal) of quantile normalised log2 H/L protein ratios for all samples of the single time point pulse-SILAC experiment. **b**, Distribution of log2 H/L protein ratios before normalisation. **c**, Distribution of log2 H/L protein ratios after quantile normalisation.

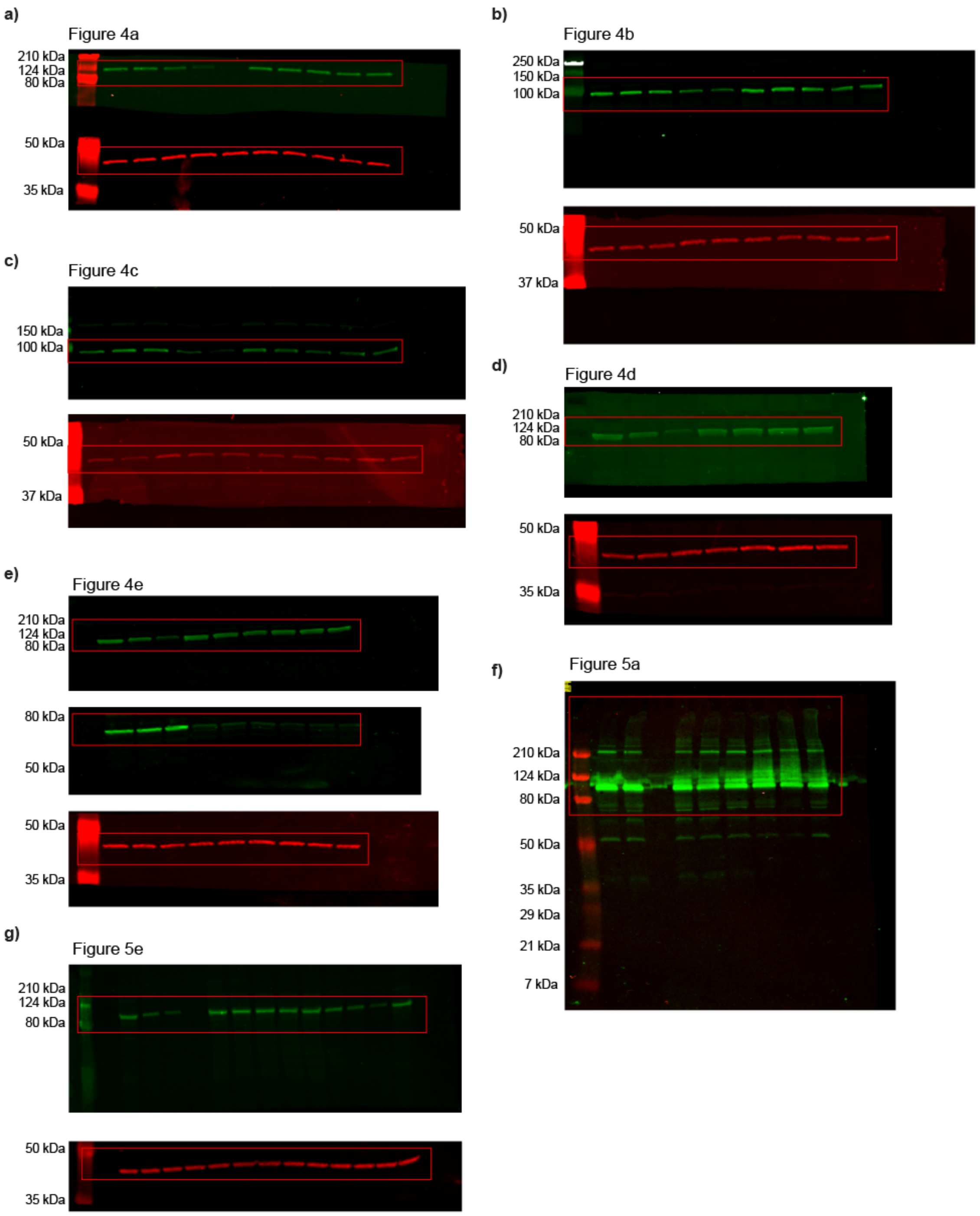

Supplementary Figure 4 | Uncropped Immunoblots. Boxed areas correspond to image regions represented in the indicated main text figures. Size marker (kDa) are indicated. **a,** Figure 4a; **b,** Figure 4b; **c,** Figure 4c; **d,** Figure 4d; **e,** Figure 4e; **f,** Figure 5a; and **g,** Figure 5e.

Supplementary Table 1 | Design matrix used for limma analysis across experiments

Experiment	Intercept	Hct116	T16	30 µM Lena
T16 DMSO1	1	0	1	0
T16 DMSO 2	1	0	1	0
T16 LENA 2	1	0	1	1
T16 LENA 1	1	0	1	1
T6 DMSO 1	1	0	0	0
T6 DMSO 2	1	0	0	0
T6 LENA 1	1	0	0	1
T6 LENA 2	1	0	0	1
Hct116 DMSO 1	1	1	1	0
Hct116 DMSO 2	1	1	1	0
Hct116 LENA 1	1	1	1	1
Hct116 LENA 2	1	1	1	1
